# Tumor biology and access to care and metastatic breast cancer outcomes

**DOI:** 10.1007/s10549-025-07881-6

**Published:** 2025-12-26

**Authors:** Matthew R. Dunn, Sarah C. Van Alsten, Marc A. Emerson, Katherine Reeder-Hayes, Terry Hyslop, Melissa A. Troester

**Affiliations:** 1 Cancer Prevention Fellowship Program, Division of Cancer Prevention, National Cancer Institute, 9609 Medical Center Drive, 5E203, Rockville, MD 20892 USA; 2 Department of Epidemiology, University of North Carolina, Chapel Hill, NC USA; 3 Lineberger Comprehensive Cancer Center, University of North Carolina, Chapel Hill, NC USA; 4 Division of Oncology, Department of Medicine, University of North Carolina School of Medicine, Chapel Hill, USA; 5 Sidney Kimmel Cancer Center, Thomas Jefferson University, Philadelphia, PA USA; 6 Department of Pathology and Laboratory Medicine, University of North Carolina, Chapel Hill, NC USA

**Keywords:** Cohort study, Disparities, Healthcare, Neighborhood, Equity

## Abstract

**Purpose:**

To understand how access to care influences metastatic breast cancer burden (MBC) while accounting for molecular tumor characteristics, and identify interventions to reduce metastatic disease burden.

**Methods:**

The Carolina Breast Cancer Study is a population-based cohort with invasive breast cancer (diagnosed 2008–2013). Both de novo metastasis (stage IV at diagnosis) and distant recurrence were evaluated (12 years of follow-up. Tumor data were from medical records, pathology reports, and RNA expression data. Social variables and access to care were from participant surveys. Generalized linear models were used to estimate associations of biological and access characteristics with MBC; Cox models were used to estimate recurrence hazards.

**Results:**

464/2998 patients (15.5%) had MBC (n = 109 de novo*;* n = 355 recurrent). MBC was associated with grade 3 vs 1 (odds ratio (OR) = 4.15, 95% CI: 2.60, 6.99), LumB vs LumA (OR = 2.08, 95% CI: 1.48, 2.90), and high vs low PAM50 risk of recurrence score (OR = 4.45, 95% CI: 2.93, 6.99) vs. non-MBC. MBC was associated with Black race (Hazard ratio (HR) = 1.66, 95% CI: 1.32, 2.11), poverty (HR = 1.47; 95% CI: 1.09, 1.99), and low education (HR = 1.48, 95% CI: 1.03, 2.13). Controlling for healthcare access (screening, regular care, delayed treatment, and community healthcare) attenuated associations with metastasis for poverty and education, but had lesser effects on race associations.

**Conclusions:**

Disparities in MBC burden persist after adjustment for individual- and community-level healthcare access. Reducing burden of MBC in Black women necessitates simultaneous targeting of biological and access to care factors.

**Supplementary Information:**

The online version contains supplementary material available at 10.1007/s10549-025-07881-6.

## Introduction

Breast cancer treatment advances have extended survival times for women with metastatic breast cancer (MBC), with one recent simulation study showing that median survival after metastatic recurrence has increased from 1.9 years to 3.2 years (averaged across 4 models) between 2000 and 2019 [1]. In the United States (USA) approximately 5–10% of breast cancer cases are stage IV at diagnosis (i.e. de novo metastatic) [2] and another 30% of breast cancer patients will eventually experience metastases (distant recurrence) [3]. The relative 5-year survival for de novo MBC is about 30% (compared to 99% for localized disease) [4], which is compounded by high care costs and quality of life decrements [5–7]. The population of survivors with MBC in the USA is projected to reach almost 250,000 by the year 2030 [8], underscoring the need to understand social and biological drivers of MBC. Metastasis detected at diagnosis (de novo) and recurrent MBC are handled very differently for clinical management. However, it is likely that inequities in healthcare access that lead to diagnostic delays (contributing to de novo metastasis) also lead to latent tumor cell dissemination and treatment failures (contributing to recurrent metastasis). Further, some patients who experience metastasis within a few years of an early stage diagnosis likely harbored circulating tumor cells at diagnosis [9], suggesting that some ‘recurrent’ metastases may actually represent asymptomatic de novo presentations. Considering metastases of both types together and separately can identify shared and distinct risk factors.

Black women are more likely to be diagnosed with MBC [10] and have decreased metastatic survival compared to White women [11]. In part, these differences in prevalence of metastatic disease reflect underlying tumor biology differences. MBC tends to be associated with more aggressive breast cancer subtypes (as defined by negative estrogen receptor status and basal-like or HER2-enriched intrinsic subtype), Ki-67 overexpression, and *BRCA1/BRCA2* mutations [12–15]. However, few studies have simultaneously evaluated tumor biologic and access to care factors in the burden of MBC. The potential for improved access to care (especially during the diagnostic window) to reduce disparities in MBC risk is understudied. One study found that 30% of Black-White differences in MBC survival were still unexplained after controlling for socioeconomic factors, tumor characteristics, and metastatic pattern, but did not include intervenable healthcare factors such as screening, primary care, and treatment timeliness [11].

We leveraged data from the population-based Carolina Breast Cancer Study (which includes overrepresentation of younger and Black women) to understand variation in MBC. Our objectives were 1) to estimate associations between molecular factors (from the diagnostic sample) and MBC; 2) to estimate disparities in MBC by race, poverty, and education, and 3) to evaluate the attenuation of MBC disparities after adjustment for molecular, healthcare, and neighborhood factors.

## Methods

### Study population

The Carolina Breast Cancer Study (CBCS) Phase III is a population-based cohort of 2998 women (age 23–74 years at diagnosis) with a first primary breast cancer diagnosis between 2008 and 2013. The CBCS enrolled participants from central and eastern North Carolina, USA and oversampled Black and younger women, attaining a study population that is half Black and half less than aged 50 years at diagnosis. Cases were identified by rapid case ascertainment from the North Carolina Central Cancer Registry. Detailed methodology for the Carolina Breast Cancer Study has been described previously [16, 17]. The study was approved by the University of North Carolina Institutional Review Board in accordance with U.S. Common Rule. All study participants provided written informed consent prior to study entry. This study complied with relevant ethical regulations, including the Declaration of Helsinki.

### Outcome assessment

Participants were defined as having MBC if they had stage IV cancer at diagnosis (de novo MBC) or if they were diagnosed with stage I-III cancer and subsequently experienced distant recurrence. Follow-up was from diagnosis date through April 2025.

### Social and clinical characteristics

Healthcare history and demographic information were collected by study nurses during in-home interviews (approximately 6 months after diagnosis). Race (N = 1495 Black and N = 1503 non-Black) was measured by participant self-classification; non-Black participants included 82 participants who did not identify as White (American Indian (N = 12), Asian, (N = 33), or another race not listed on the survey (N = 37)). We interpret race as a social construct, distinct from genetic ancestry [18]. Poverty status was determined based on self-reported household income and household size [19]. Self-reported education was binarized as less than high school and high school or more, based on observation that differences in MBC were most prominent at this threshold. Participants reported ‘regular care’ (a source of regular healthcare, including primary and specialty care, in the 10 years prior to diagnosis) and pre-diagnostic mammography history (to classify participants as adherent/non-adherent to biennial screening) as described in Dunn et al. [20]. We included a community-level measure of healthcare access linked to participant home residence at diagnosis [21]. For this measure, North Carolina census tracts were characterized by area-level healthcare affordability and geographic accessibility characteristics.

Tumor AJCC stage, size, and grade were obtained from participant medical records, as were dates of diagnosis, initial treatment, and distant recurrence. Delayed treatment was defined as greater than 30 days between confirmed diagnosis and treatment initiation; this threshold was used in prior CBCS work and is based on literature showing decrements in survival for delays beyond 30 days [22, 23].

### Molecular data

Estrogen receptor (ER) status, progesterone receptor (PR) status, and human epidermal growth factor receptor 2 (HER2) status were obtained from pathology reports; ER and PR positivity were defined as ≥ 1% staining by immunohistochemistry (IHC), and HER2 positivity was 3 + by IHC or FISH assay. IHC-based subtype (Luminal A, Luminal B, ER-/HER2 + , Basal-like) was classified based on ER, PR, and HER2 status as previously described [24].

For RNA-based signatures, we measured expression of 219 subtype, proliferation, and immune-related genes on the NanoString platform. As previously described [25], expression data were normalized by applying removal of unwanted variation (the RUVg function) from the RUVseq package [26] to correct for batch effects and technical variation, and all gene expression values were log2 transformed and median centered. In total, 1969 participants in CBCS3 had gene expression assessed (N = 1,646 primary tumors from those who developed MBC, N = 323 without). We applied the 50-gene PAM50 classifier to determine molecular subtype and risk of recurrence score with proliferation (ROR-P) and tumor size (ROR-PT) [27]. Latent class analysis of 48 immune genes identified three patterns of immune response in the CBCS: adaptive-enriched, innate-enriched, and immune-quiet [28]. RNA-based expression of DNA repair genes was used to classify participants with homologous recombination deficiency (HRD-high), also described previously [29]. Finally, we classified tumors with dysfunctional p53 (“Mutant-like”) using expression of 48 genes according to previous methods [30].

### Statistical analysis

Associations between molecular characteristics and metastasis were estimated using logistic regression. We report odds ratios (ORs) and 95% confidence intervals (CIs) for MBC vs. no MBC, unadjusted and adjusted for delayed treatment initiation (a proxy for individual access and receipt of timely cancer care) and community-level healthcare access. MBC was considered overall and stratified on de novo vs. recurrent.

Among screening-age participants (age 45 + , N = 2061), we evaluated MBC in association with race, poverty, and education. Linear-risk regression was used to estimate prevalence differences (PDs) and 95% CIs. We reported unadjusted models and multivariable models that adjust for access factors (screening, regular care, community affordability and accessibility). We interpret these serial models as approximations of the difference in metastatic cancer prevalence that would occur if access to care and/or triple-negative status were equalized. Associations with poverty and education were also estimated separately for Black and non-Black participants.

Finally, we assessed risk of metastasis over time using the Kaplan–Meier estimator and Cox proportional hazards regression. Differences by race, poverty, and education were assessed with incidence curves and Hazard Ratios (HRs) with 95% CIs. Models were computed as unweighted (representing the observed disparity) and with inverse probability weights (IPW) that incorporated screening, regular care, delayed treatment, and community healthcare affordability/accessibility. The IPW analysis was performed with the ‘weightit’ R package and used stabilized average treatment effect (ATE) weights [31]. In a sensitivity analysis, we repeated weighting and time-to-event analyses among participants with ER + disease only. Data analysis and visualization was performed in R Studio Version 12.0 (R Foundation for Statistical Computing; Vienna, Austria).

## Results

The combined prevalence of de novo and recurrent MBC was 15.5% (Table 1). Compared to participants without MBC, the prevalence of MBC was greater among participants identifying as Black vs non-Black (19% vs 12%), living below the federal poverty level (21% vs 14%), having less than high school education (21% vs 15%), and lacking pre-diagnostic regular care (22% vs 15%). Among screening-age participants, MBC was more frequent among those non-adherent to biennial screening compared to adherent (21% vs 11%).

**Table 1:** Carolina breast cancer study participant characteristics by metastasis status (N = 2998)

	Non-metastatic	Metastatic^a^
	N = 2,534	N = 464
	N (%)	N (%)
Race		
non-Black	1319 (88%)	184 (12%)
Black	1215 (81%)	280 (19%)
Age		
< 50 years	1239 (83%)	253 (17%)
50 + years	1295 (86%)	211 (14%)
ER Status		
Negative	712 (80%)	183 (20%)
Positive	1814 (87%)	280 (13%)
HER2 Status		
Negative	2148 (85%)	387 (15%)
Positive	374 (83%)	77 (17%)
Poverty Status		
No	2024 (86%)	331 (14%)
Yes	382 (79%)	99 (21%)
Missing	128	34
Education		
High school (HS) or more	2345 (85%)	413 (15%)
< HS	189 (79%)	50 (21%)
Regular care^b^		
Regular care	2,224 (85%)	378 (15%)
No Regular Care	310 (78%)	86 (22%)
Screening status^c^		
Screened	1229 (89%)	146 (11%)
Under-screened	540 (79%)	143 (21%)
< 45 years of age or missing	765	175
Treatment delay^d^		
No	1,640 (84%)	302 (16%)
Yes	891 (85%)	162 (15%)
Community healthcare access^e^		
High affordability, low accessibility	892 (87%)	139 (13%)
High affordability, low accessibility	772 (85%)	137 (15%)
Low affordability, high accessibility	394 (83%)	80 (17%)
Low affordability, low accessibility	476 (82%)	108 (18%)

^a^Includes n = 109 de novo (i.e., stage IV at diagnosis*)* and n = 355 recurrent metastases

^b^Presence/absence of primary care before diagnosis

^c^Defined as receipt of at least one mammogram every 2 years

^d^Delayed treatment initiation defined as more than 30 days between confirmed diagnosis and first treatment

^e^Community access reflects census tract classifications based on healthcare affordability and accessibility characteristics

### Molecular characteristics of MBC

Associations between tumor molecular characteristics and metastasis (among N = 1969 participants with available RNA data) are shown in Table 2. MBC was more prevalent among participants with more aggressive tumor features, including grade 3 vs grade 1 (OR = 4.15, 95% CI: 2.60, 6.99), LumB vs LumA (OR = 2.08, 95% CI: 1.48, 2.90), HER2-encriched vs LumA (OR = 2.13, 95% CI: 1.45, 3.12), Basal-like vs LumA (OR = 1.42, 95% CI: 1.03, 1.96), mutant p53 vs wildtype (OR = 1.45, 95% CI: 1.14, 1.84), and HRD-high vs -low (OR = 1.61, 95% CI: 1.27, 2.05), high vs low ROR-PT (OR = 4.45, 95% CI: 2.93, 6.99). MBC was less frequent among participants with adaptive vs quiet immune response (OR = 0.50, 95% CI: 0.35, 0.69). These ORs were largely unchanged after adjusting for delayed care and community-level healthcare access. We also performed sensitivity analyses excluding de novo metastases, and associations were consistent in direction and retained statistical significance, with small differences in magnitude. These recurrence-only associations were also unchanged after adjustment for community healthcare access and delayed care.

**Table 2:** Associations between tumor molecular characteristics and metastasis, among participants with RNA data (N = 1969)

	No Metastasis	Metastasis	OR (95% CI)	Adj OR (95% CI)^a^	OR (95% CI)	Adj OR (95% CI)^a^
	N = 1646	N = 323	Any Metastasis	Any Metastasis	Recurrent Only^b^	Recurrent Only^b^
**Grade**						
1	320 (94.4)	< 20	REF	REF	REF	REF
2	594 (83.5)	117 (16.5)	3.32 (2.05, 5.65)	3.30 (2.04, 5.62)	2.82 (1.69, 4.98)	2.81 (1.68, 4.96)
3	719 (80.2)	177 (19.8)	4.15 (2.60, 6.99)	4.07 (2.55, 6.86)	3.67 (2.24, 6.38)	3.61 (2.20, 6.29)
**PAM50**						
LumA	592 (87.3)	86 (12.7)	REF	REF	REF	REF
LumB	272 (76.8)	82 (23.2)	2.08 (1.48, 2.90)	2.05 (1.46, 2.87)	1.91 (1.30, 2.80)	1.90 (1.29, 2.77)
Her2	171 (76.3)	53 (23.7)	2.13 (1.45, 3.12)	2.12 (1.44, 3.10)	1.99 (1.28, 3.06)	1.99 (1.28, 3.05)
Basal	426 (82.9)	88 (17.1)	1.42 (1.03, 1.96)	1.38 (1.00, 1.91)	1.68 (1.19, 2.39)	1.65 (1.16, 2.35)
Normal	185 (93.0)	< 20	0.52 (0.28, 0.91)	0.53 (0.28, 0.92)	0.53 (0.26, 0.99)	0.54 (0.26, 1.00)
**IHC Subtype**						
LumA	577 (89.3)	69 (10.7)	REF	REF	REF	REF
LumB	456 (79.3)	119 (20.7)	2.18 (1.59, 3.02)	2.22 (1.61, 3.07)	1.94 (1.36, 2.79)	1.96 (1.37, 2.81)
ER-/HER2 +	93 (83.0)	< 20	1.71 (0.96, 2.92)	1.76 (0.99, 3.02)	1.33 (0.66, 2.49)	1.35 (0.67, 2.54)
Basal	355 (81.2)	82 (18.8)	1.93 (1.37, 2.74)	1.91 (1.35, 2.71)	2.06 (1.42, 3.01)	2.04 (1.40, 2.98)
**P53**						
WT-like	962 (85.8)	159 (14.2)	REF	REF	REF	REF
Mut-like	684 (80.7)	164 (19.3)	1.45 (1.14, 1.84)	1.41 (1.11, 1.79)	1.56 (1.20, 2.03)	1.53 (1.17, 2.00)
**HRD**						
HRD Low	989 (86.4)	156 (13.6)	REF	REF	REF	REF
HRD High	657 (79.7)	167 (20.3)	1.61 (1.27, 2.05)	1.58 (1.25, 2.02)	1.75 (1.34, 2.29)	1.72 (1.32, 2.26)
**ROR-PT**						
Low	418 (93.9)	27 (6.1)	REF	REF	REF	REF
Intermediate	748 (83.1)	152 (16.9)	3.15 (2.09, 4.91)	3.08 (2.04, 4.82)	3.50 (2.18, 5.94)	3.46 (2.15, 5.87)
High	480 (77.7)	138 (22.3)	4.45 (2.93, 6.99)	4.31 (2.83, 6.78)	5.22 (3.23, 8.90)	5.12 (3.16, 8.72)
**Immune Class**						
Quiet	696 (81.6)	157 (18.4)	REF	REF	REF	REF
Innate	478 (80.9)	113 (19.1)	1.05 (0.80, 1.37)	1.04 (0.79, 1.35)	0.99 (0.73, 1.33)	0.98 (0.73, 1.32)
Adaptive	472 (89.9)	53 (10.1)	0.50 (0.35, 0.69)	0.48 (0.34, 0.67)	0.44 (0.30, 0.63)	0.43 (0.29, 0.62)

There were 23 participants missing information on grade, 6 missing ROR-PT, and 199 missing IHC Subtype

^a^Adjusted for delayed treatment initiation and community-level healthcare access (affordability and accessibility)

^b^Recurrent-only odds ratios (ORs) compare individuals with a recurrent metastasis (i.e., not Stage IV at diagnosis) to those who never developed metastasis, and include 1899 participants

### Social disparities in MBC

Table 3 shows that MBC was more prevalent among Black vs non-Black participants, participants with income below the poverty level, and less educated participants. The association of MBC with race (PD = 6.2%, 95% CI: 3.2, 9.2) was only slightly attenuated after adjustment for triple-negative status (PD = 5.4%, 95% CI: 2.4, 8.4). Adjustment for access factors (regular care, screening, and community-level access) reduced the PD (95% CI) to 4.3% (1.2, 7.4), meaning that about two-thirds of the observed race difference was unexplained after adjusting for these factors. The excess MBC associated with poverty was lower and not significant (PD = 4.4%, 95% CI: -0.1, 9.0), and was unaffected by adjustment for triple-negative status. However, in contrast to results for race, controlling for access factors moved the poverty-MBC association substantially toward the null. After adjustment for regular care, screening, and community access the PD was 1.2% (95% CI: -3.5, 5.9). Adjustment for access factors and triple-negative status further shifted the estimate toward the null (PD = 0.7%, 95% CI: -4.0, 5.3). Education showed similar patterns to poverty. Participants without a high school diploma had higher MBC frequency (PD = 5.0%, 95% CI: -0.9, 11.0), which was shifted toward the null after adjustment for access factors (PD = 1.2%, 95% CI: -5.0, 7.3). In race-stratified analyses, patterns were similar for Black and non-Black participants. Unadjusted associations of poverty and education with MBC were smaller in magnitude (compared to all participants) and non-significant, and the point estimates were reduced almost to 0 after adjustment for screening and regular care.

**Table 3:** Metastasis frequency by race, poverty, and education after adjustment for molecular and access factors, among participants age 45 + (N = 2062)

		Model 1	Model 2	Model 3	Model 4		Model 5
	Metastasis frequency	Unadjusted	+ TN status	+ Screening,^a^ regular care^b^	+ Screening,^a^ regular care,^b^ community access^3^	+ screening,^a^ regular care,^b^ community access,^3^ TN status,^4^	
Race	N (%)	PD (95% CI)	PD (95% CI)	PD (95% CI)	PD (95% CI)	PD (95% CI)	
non-Black	108 (11%)	0 (ref)	0 (ref)	0 (ref)	0 (ref)	0 (ref)	
Black	173 (17%)	6.2 (3.2, 9.2)	5.4 (2.4, 8.4)	4.7 (1.8, 7.7)	5.3 (2.2, 8.4)	4.3 (1.2, 7.4)	
Poverty Status							
No	212 (12%)	0 (ref)	0 (ref)	0 (ref)	0 (ref)	0 (ref)	
Yes	53 (17%)	4.4 (-0.1, 9.0)	4.2 (-0.3, 8.7)	1.3 (-3.4, 5.9)	1.2 (-3.5, 5.9)	0.7 (-4.0, 5.3)	
Education							
HS or more	256 (13%)	0 (ref)	0 (ref)	0 (ref)	0 (ref)	0 (ref)	
< HS	33 (18%)	5.0 (-0.9, 11.0)	5.4 (-0.4, 11.3)	1.1 (-5.0, 7.2)	1.2 (-5.0, 7.3)	1.6 (-4.4, 7.7)	
		Black					
Poverty Status							
No	113 (16%)	0 (ref)	0 (ref)	0 (ref)	0 (ref)	0 (ref)	
Yes	45 (19%)	2.5 (-3.1, 8.1)	2.6 (-3.0, 8.1)	-0.5 (-6.1, 5.1)	-0.4 (-6.0, 5.3)	-0.6 (-6.2, 5.0)	
Education							
HS or more	147 (17%)	0 (ref)	0 (ref)	0 (ref)	0 (ref)	0 (ref)	
< HS	26 (21%)	4.1 (-3.4, 11.6)	4.5 (-3.0, 12.0)	0.6 (-7.1, 8.3)	1.0 (-6.7, 8.6)	1.2 (-6.4, 8.9)	
		Non-Black					
Poverty Status							
No	99 (11%)	0 (ref)	0 (ref)	0 (ref)	0 (ref)	0 (ref)	
Yes	8 (13%)	2.3 (-6.3, 10.9)	2.5 (-5.9, 11.0)	-0.1 (-8.7, 8.4)	0.2 (-8.2, 8.7)	0.4 (-7.7, 8.5)	
Education							
HS or more	109 (11%)	0 (ref)	0 (ref)	0 (ref)	0 (ref)	0 (ref)	
< HS	7 (13%)	2.6 (-6.9. 12.0)	3.6 (-5.8, 13.0)	-0.3 (-10.0, 9.4)	0.2 (-9.3, 9.8)	1.6 (-7.8, 11.0)	

^a^Screening adherence was defined by receipt of at least one mammogram every 2 years

^b^Regular care refers to the presence/absence of primary care before diagnosis

^c^Community access reflects census tract classifications based on healthcare affordability and accessibility characteristics

^d^TN = triple-negative

**Fig. 1:**
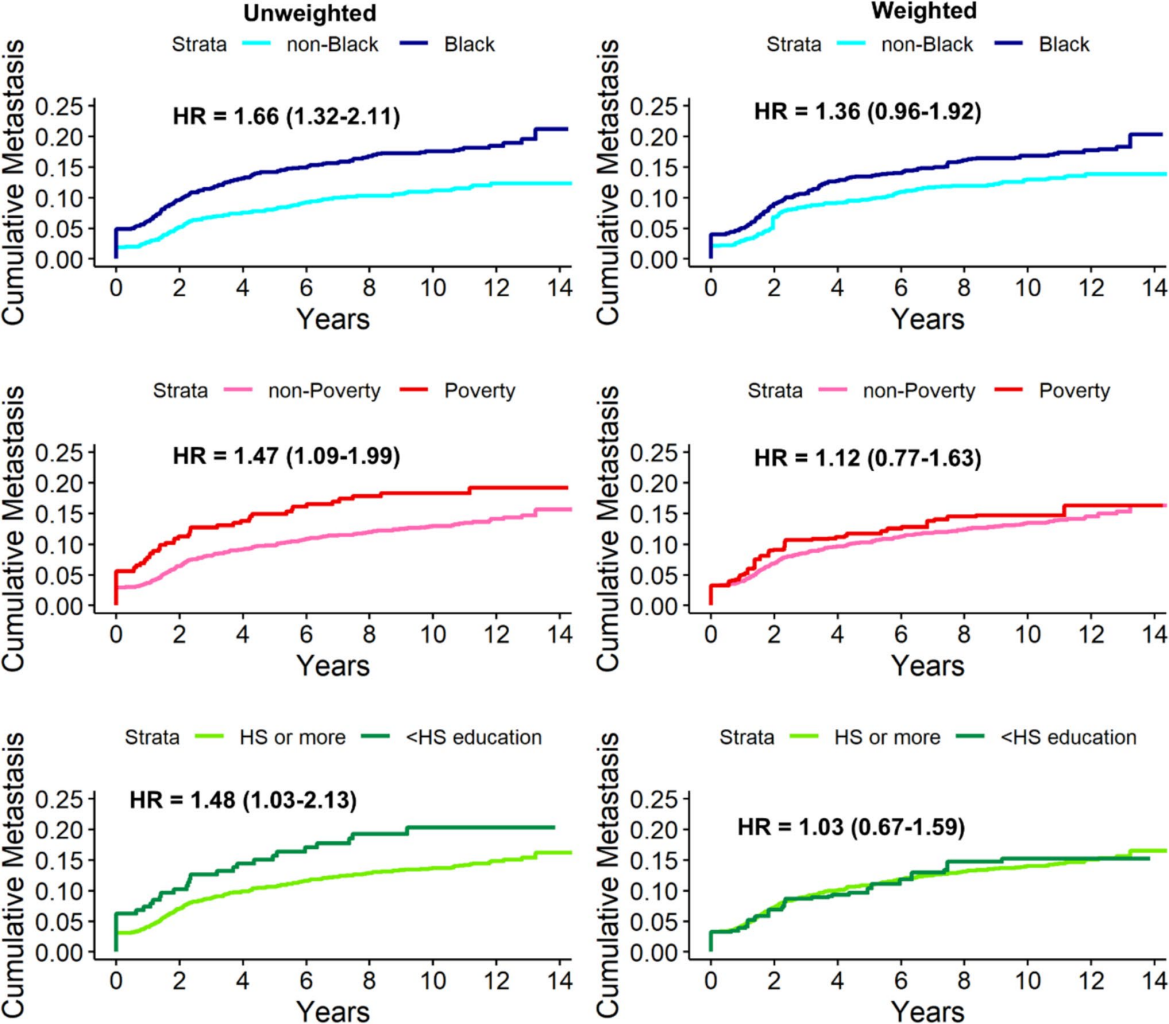
Cumulative incidence of metastasis with weighting for healthcare access factors (screening, regular care, delayed treatment initiation, and community healthcare access)

We also evaluated the impact of healthcare access on MBC disparities with IPW-weighted time-to-event analyses (Fig. 1). The unweighted HR (95% CI) for Black race was 1.66 (1.32, 2.11) and shifted modestly toward the null after weighting for screening, regular care, delayed treatment, and community-level access (HR = 1.36, 95% CI: 0.96, 1.92). In contrast, the unweighted estimates for poverty (HR = 1.47; 95% CI: 1.09, 1.99) and education (HR = 1.48, 95% CI: 1.03–2.13) shifted to the null in the weighted models (poverty HR = 1.12, 95% CI: 0.77, 1.63 and education HR = 1.03, 95% CI: 0.67–1.59). In a sensitivity analysis restricted to ER + participants, the HRs were slightly greater in magnitude, but the overall pattern was consistent: associations of poverty and education with MBC shifted to the null after weighting, while the association of race with MBC had substantial unexplained variation (Supplemental Fig. 1).

Cox proportional hazards models were used to model metastasis incidence using unweighted and inverse probability weighted (IPW) models with average treatment effect weights. Models were weighted for screening, regular care, delayed treatment initiation, and community healthcare access. Analysis restricted to n = 2,062 patients aged 45 years and older at diagnosis.

## Discussion

We assessed inequities in MBC by race, poverty, and education and found the strongest differences by race. Controlling for individual- and community-level healthcare access shifted poverty- and education-associated differences toward the null. However, adjusting for these same factors left substantial unexplained differences between Black and non-Black participants, suggesting that racial differences in MBC are driven by a more complex set of factors than just SES or healthcare access. For instance, we found that aggressive molecular tumor features present at diagnosis were strongly associated with MBC. Still, controlling for triple-negative subtype, which is more prevalent among Black women compared to White women [32], did not meaningfully alter racial differences in MBC.

Associations of MBC with tumor molecular characteristics were not attenuated after adjustment for access factors, suggesting that the critical intervention window for intercepting tumor biology-associated MBC exists earlier in cancer development (i.e. is largely unaffected by diagnostic patterns and cancer care variables). This compounds the observation that Black-White disparities are often greatest for the most treatable cancers [33]. There may be etiologic factors that differ in frequency leading to excess biologically mediated MBC in black women. A recent scoping review identified parity, age at first birth, and post-menopausal body mass index (BMI) as potential drivers of etiologic heterogeneity, or differential risk of specific subtypes, in IHC-defined ER + versus ER- breast cancers [34]. Other studies have suggested these factors as contributors to the disproportionate burden of aggressive subtype in Black women [35, 36]. Combining our results with prior literature, it seems that Black women experience both an excess burden of aggressive subtypes that are relatively intractable to health-care interventions that avoid metastasis in other groups, and also experience greater burden of aggressive subtypes associated with metastasis. In our study, MBC was about 50% more prevalent among Black compared to non-Black women (18.7% vs 12.2%).

Community-level factors are another potential modifiable etiologic factor, with several studies showing differences in aggressive IHC-based subtype prevalence by neighborhood SES and segregation, both overall and among Black women only [37–39]. It remains unclear whether similar associations exist for RNA-based subtypes, and whether interventions on etiologic factors can narrow the gap on racial disparities in MBC survival. Most studies have focused on the post-diagnostic window, including several showing that breast cancer survival is associated with neighborhood-level deprivation [40–43]. Such studies typically measure SES around the time of diagnosis, although one analysis showed that lower early-life socioeconomic position was associated with higher breast cancer mortality [44]. Overall, however, few studies have considered early-life SES or other etiologic factors in association with MBC.

A limitation of our analysis is that we did not conduct a formal mediation analysis; however, we were interested in assessing variation in burden and not in estimating causal effects. Moreover, because MBC is rare (15% at 12 years) we did not have power to conduct a mediation analysis. However, a strength of our analysis is having both detailed molecular data and social data on the same population, allowing us to consider both factors in a more integrated way. Another potential limitation was the aggregation of de novo and recurrent MBC. Since de novo metastases have a greater share of aggressive tumor subtypes, this group may be less responsive to healthcare interventions, which would attenuate the associations relative to what we observed. Also, RNA data that was only available for about two-thirds of the overall study population, so we lacked complete molecular and access data on the full complement of participants. Tumors that passed RNA quality control also tended to be slightly larger and higher grade than those without RNA and thus estimates may not represent the full spectrum of earlier-stage tumors present. However, the population-based CBCS includes greater frequency of lower stage and small tumors compared to many molecularly detailed datasets like the TCGA. We were also unable to control for treatment adherence or long-term maintenance therapy which affect risk of distant recurrence. We had too few participants with ER + /HER2- disease to assess the contribution of endocrine adherence to metastasis by race, poverty, and education. However, prior literature has shown that Black and lower-SES women have lower adherence to chemotherapy and endocrine therapy [45, 46]. Finally, results may not be fully generalizable to populations beyond the CBCS (which oversampled Black and younger women) and North Carolina, USA.

The central aim of this work was to better understand the social and biologic drivers of MBC, including de novo and recurrent metastases. Prior studies of MBC that included both types of metastasis have primarily evaluated survival outcomes, reporting lower survival for Black women [11, 47], patients with low individual SES [11], and patients living in neighborhoods with high deprivation [47] and segregation [48]. Our study adds to this literature by assessing the factors associated with metastasis and assessing unexplained variation in metastasis after controlling for multiple factors. Our findings suggest that differences in MBC frequency may be tractable for those in poverty and with low education by providing high-quality care. However, unexplained variation among Black women continues to be a high priority. Interventions targeting the etiology of aggressive breast cancer subtypes offer one priority focus for metastasis prevention. Disparities in treatment patterns and quality of care throughout survivorship are also impactful and worthy of consideration. As the number of women living with MBC is projected to increase in the coming decades [8, 49, 50], understanding unmet needs (including management of comorbidities that are associated with metastatic disease [51]) and defining novel preventive interventions are of critical importance for breast cancer control.

## Supplementary Information

Below is the link to the electronic supplementary material.

Supplementary file1 (DOCX 357 KB)

## Data Availability

The data that support the findings of this study are available upon submission of a letter of intent and approval from the Carolina Breast Study Steering Committee (https://ciphr.unc.edu/cbcs-loi-form.php) and IRB approval. The data are not publicly available to protect privacy of study participants.

## References

[1] Caswell-Jin JL, Sun LP, Munoz D et al (2024) Analysis of breast cancer mortality in the US—1975 to 2019. JAMA 331:233–241. 10.1001/jama.2023.25881 38227031 10.1001/jama.2023.25881 PMC10792466

[2] CDC (2025) Metastatic Female Breast Cancer Incidence. In: U. S. Cancer Stat. https://www.cdc.gov/united-states-cancer-statistics/publications/metastatic-breast-cancer.html. Accessed 9 Apr 2025

[3] Redig AJ, McAllister SS (2013) Breast cancer as a systemic disease: a view of metastasis. J Intern Med 274:113–126. 10.1111/joim.12084 23844915 10.1111/joim.12084 PMC3711134

[4] American Cancer Society Cancer Facts and Figures 2024. https://www.cancer.org/research/cancer-facts-statistics/all-cancer-facts-figures/2024-cancer-facts-figures.html. Accessed 2 Mar 2024

[5] Castel LD, Abernethy AP, Li Y et al (2007) Hazards for pain severity and pain interference with daily living, with exploration of brief pain inventory cutpoints, among women with metastatic breast cancer. J Pain Symptom Manage 34:380–392. 10.1016/j.jpainsymman.2006.12.007 17583467 10.1016/j.jpainsymman.2006.12.007

[6] Hamer J, McDonald R, Zhang L et al (2017) Quality of life (QOL) and symptom burden (SB) in patients with breast cancer. Support Care Cancer 25:409–419. 10.1007/s00520-016-3417-6 27696078 10.1007/s00520-016-3417-6

[7] Reed E, Simmonds P, Haviland J, Corner J (2012) Quality of life and experience of care in women with metastatic breast cancer: a cross-sectional survey. J Pain Symptom Manage 43:747–758. 10.1016/j.jpainsymman.2011.05.005 22088804 10.1016/j.jpainsymman.2011.05.005

[8] Gogate A, Wheeler SB, Reeder-Hayes KE et al (2021) Projecting the prevalence and costs of metastatic breast cancer from 2015 through 2030. JNCI Cancer Spectr 5:pkab063. 10.1093/jncics/pkab063 34409255 10.1093/jncics/pkab063 PMC8364673

[9] Riggio AI, Varley KE, Welm AL (2021) The lingering mysteries of metastatic recurrence in breast cancer. Br J Cancer 124:13–26. 10.1038/s41416-020-01161-4 33239679 10.1038/s41416-020-01161-4 PMC7782773

[10] Giaquinto AN, Sung H, Newman LA et al (2024) Breast cancer statistics 2024. CA Cancer J Clin 74:477–495. 10.3322/caac.21863 39352042 10.3322/caac.21863

[11] Ren J-X, Gong Y, Ling H et al (2019) Racial/ethnic differences in the outcomes of patients with metastatic breast cancer: contributions of demographic, socioeconomic, tumor and metastatic characteristics. Breast Cancer Res Treat 173:225–237. 10.1007/s10549-018-4956-y 30293212 10.1007/s10549-018-4956-y PMC6394580

[12] Bai X, Ni J, Beretov J et al (2021) Triple-negative breast cancer therapeutic resistance: where is the Achilles’ heel? Cancer Lett 497:100–111. 10.1016/j.canlet.2020.10.016 33069769 10.1016/j.canlet.2020.10.016

[13] Esteva FJ, Sahin AA, Cristofanilli M et al (2002) Molecular prognostic factors for breast cancer metastasis and survival. Semin Radiat Oncol 12:319–328. 10.1053/srao.2002.35251 12382190 10.1053/srao.2002.35251

[14] Yang H, Wang R, Zeng F et al (2020) Impact of molecular subtypes on metastatic behavior and overall survival in patients with metastatic breast cancer: a single‑center study combined with a large cohort study based on the Surveillance, Epidemiology and End Results database. Oncol Lett 20:87. 10.3892/ol.2020.11948 32863920 10.3892/ol.2020.11948 PMC7436893

[15] Song Y, Barry WT, Seah DS et al (2020) Patterns of recurrence and metastasis in BRCA1/*BRCA2*-associated breast cancers. Cancer 126:271–280. 10.1002/cncr.32540 31581314 10.1002/cncr.32540 PMC7003745

[16] Emerson MA, Reeder-Hayes KE, Tipaldos HJ et al (2020) Integrating biology and access to care in addressing breast cancer disparities: 25 years’ research experience in the Carolina Breast Cancer Study. Curr Breast Cancer Rep 12:149–160. 10.1007/s12609-020-00365-0 33815665 10.1007/s12609-020-00365-0 PMC8011652

[17] Newman B, Moorman PG, Millikan R et al (1995) The Carolina Breast Cancer Study: integrating population-based epidemiology and molecular biology. Breast Cancer Res Treat 35:51–60. 10.1007/BF00694745 7612904 10.1007/BF00694745

[18] Smedley BD (2012) The lived experience of race and its health consequences. Am J Public Health 102:933–935. 10.2105/AJPH.2011.300643 22420805 10.2105/AJPH.2011.300643 PMC3483928

[19] U.S. Department of Health and Human Services, Assistant Secretary for Planning and Evaluation (ASPE) (2024) Poverty Guidelines. In: ASPE. https://aspe.hhs.gov/topics/poverty-economic-mobility/poverty-guidelines. Accessed 10 Aug 2024

[20] Dunn MR, Metwally EM, Vohra S et al (2024) Understanding mechanisms of racial disparities in breast cancer: an assessment of screening and regular care in the Carolina Breast Cancer Study. Cancer Causes Control. 10.1007/s10552-023-01833-5 38217760 10.1007/s10552-023-01833-5 PMC11045315

[21] Dunn MR, Niu H, Li D et al (2025) Applying a novel measure of community-level healthcare access to assess breast cancer care timeliness. Cancer Epidemiol Biomarkers Prev. 10.1158/1055-9965.EPI-25-0011 40298936 10.1158/1055-9965.EPI-25-0011 PMC12213174

[22] Dunn MR, Li D, Emerson MA et al (2024) A latent class assessment of healthcare access factors and disparities in breast cancer care timeliness. PLoS Med 21:e1004500. 10.1371/journal.pmed.1004500 39621782 10.1371/journal.pmed.1004500 PMC11649116

[23] Hanna TP, King WD, Thibodeau S et al (2020) Mortality due to cancer treatment delay: systematic review and meta-analysis. BMJ 371:m4087. 10.1136/bmj.m4087 33148535 10.1136/bmj.m4087 PMC7610021

[24] Allott EH, Cohen SM, Geradts J et al (2016) Performance of Three-Biomarker immunohistochemistry for intrinsic breast cancer subtyping in the AMBER Consortium. Cancer Epidemiol Biomarkers Prev 25:470–478. 10.1158/1055-9965.EPI-15-0874 26711328 10.1158/1055-9965.EPI-15-0874 PMC4779705

[25] Bhattacharya A, Hamilton AM, Furberg H et al (2021) An approach for normalization and quality control for NanoString RNA expression data. Brief Bioinform 22:bbaa163. 10.1093/bib/bbaa163 32789507 10.1093/bib/bbaa163 PMC8138885

[26] Risso D, Ngai J, Speed TP, Dudoit S (2014) Normalization of RNA-seq data using factor analysis of control genes or samples. Nat Biotechnol 32:896–902. 10.1038/nbt.2931 25150836 10.1038/nbt.2931 PMC4404308

[27] Parker JS, Mullins M, Cheang MCU et al (2009) Supervised risk predictor of breast cancer based on intrinsic subtypes. J Clin Oncol Off J Am Soc Clin Oncol 27:1160–1167. 10.1200/JCO.2008.18.1370 10.1200/JCO.2008.18.1370 PMC2667820 19204204

[28] Hamilton AM, Hurson AN, Olsson LT et al (2022) The landscape of immune microenvironments in racially diverse breast cancer patients. Cancer Epidemiol Biomarkers Prev 31:1341–1350. 10.1158/1055-9965.EPI-21-1312 35437570 10.1158/1055-9965.EPI-21-1312 PMC9292136

[29] Walens A, Van Alsten SC, Olsson LT et al (2022) RNA-based classification of homologous recombination deficiency in racially diverse patients with breast cancer. Cancer Epidemiol Biomarkers Prev 31:2136–2147. 10.1158/1055-9965.EPI-22-0590 36129803 10.1158/1055-9965.EPI-22-0590 PMC9720427

[30] Troester MA, Herschkowitz JI, Oh DS et al (2006) Gene expression patterns associated with p53 status in breast cancer. BMC Cancer 6:276. 10.1186/1471-2407-6-276 17150101 10.1186/1471-2407-6-276 PMC1764759

[31] Robins JM, Hernán MA, Brumback B (2000) Marginal structural models and causal inference in epidemiology. Epidemiol Camb Mass 11:550–560. 10.1097/00001648-200009000-00011 10.1097/00001648-200009000-00011 10955408

[32] Kohler BA, Sherman RL, Howlader N et al (2015) Annual report to the nation on the status of cancer, 1975-2011, featuring incidence of breast cancer subtypes by race/ethnicity, poverty, and state. J Natl Cancer Inst 107:djv048. 10.1093/jnci/djv048 25825511 10.1093/jnci/djv048 PMC4603551

[33] Warner ET, Tamimi RM, Hughes ME et al (2015) Racial and ethnic differences in breast cancer survival: mediating effect of tumor characteristics and sociodemographic and treatment factors. J Clin Oncol 33:2254–2261. 10.1200/JCO.2014.57.1349 25964252 10.1200/JCO.2014.57.1349 PMC4486344

[34] Hurson AN, Ahearn TU, Koka H et al (2024) Risk factors for breast cancer subtypes by race and ethnicity: a scoping review. J Natl Cancer Inst 116:1992–2002. 10.1093/jnci/djae172 39018167 10.1093/jnci/djae172 PMC11630539

[35] Palmer JR, Viscidi E, Troester MA et al (2014) Parity, lactation, and breast cancer subtypes in African American women: results from the AMBER consortium. J Natl Cancer Inst 106:dju237. 10.1093/jnci/dju237 25224496 10.1093/jnci/dju237 PMC4271113

[36] Chollet-Hinton L, Olshan AF, Nichols HB et al (2017) Biology and etiology of young-onset breast cancers among premenopausal African American women: results from the AMBER consortium. Cancer Epidemiol Biomarkers Prev 26:1722–1729. 10.1158/1055-9965.EPI-17-0450 28903991 10.1158/1055-9965.EPI-17-0450 PMC5903207

[37] Qin B, Babel RA, Plascak JJ et al (2021) Neighborhood social environmental factors and breast cancer subtypes among Black women. Cancer Epidemiol Biomarkers Prev 30:344–350. 10.1158/1055-9965.EPI-20-1055 33234556 10.1158/1055-9965.EPI-20-1055 PMC7867587

[38] Aoki R-LF, Uong SP, Gomez SL et al (2021) Individual- and neighborhood-level socioeconomic status and risk of aggressive breast cancer subtypes in a pooled cohort of women from Kaiser Permanente Northern California. Cancer 127:4602–4612. 10.1002/cncr.33861 34415571 10.1002/cncr.33861 PMC8997171

[39] Linnenbringer E, Geronimus AT, Davis KL et al (2020) Associations between breast cancer subtype and neighborhood socioeconomic and racial composition among Black and White women. Breast Cancer Res Treat 180:437–447. 10.1007/s10549-020-05545-1 32002766 10.1007/s10549-020-05545-1 PMC7066090

[40] Chen JC, Handley D, Elsaid MI et al (2024) Persistent neighborhood poverty and breast cancer outcomes. JAMA Netw Open 7:e2427755. 10.1001/jamanetworkopen.2024.27755 39207755 10.1001/jamanetworkopen.2024.27755 PMC11362869

[41] Goel N, Hernandez A, Thompson C et al (2023) Neighborhood disadvantage and breast cancer–specific survival. JAMA Netw Open 6:e238908. 10.1001/jamanetworkopen.2023.8908 37083666 10.1001/jamanetworkopen.2023.8908 PMC10122178

[42] Holder EX, Barnard ME, Xu NN et al (2025) Neighborhood disadvantage, individual experiences of racism, and breast cancer survival. JAMA Netw Open 8:e253807. 10.1001/jamanetworkopen.2025.3807 40193073 10.1001/jamanetworkopen.2025.3807 PMC11976487

[43] Cheng E, Soulos PR, Irwin ML et al (2021) Neighborhood and individual socioeconomic disadvantage and survival among patients with nonmetastatic common cancers. JAMA Netw Open 4:e2139593. 10.1001/jamanetworkopen.2021.39593 34919133 10.1001/jamanetworkopen.2021.39593 PMC8683967

[44] Akinyemiju T, Moore JX, Ojesina AI et al (2016) Racial disparities in individual breast cancer outcomes by hormone-receptor subtype, area-level socio-economic status and healthcare resources. Breast Cancer Res Treat 157:575–586. 10.1007/s10549-016-3840-x 27255533 10.1007/s10549-016-3840-x PMC4912843

[45] Roberts MC, Wheeler SB, Reeder-Hayes K (2015) Racial/ethnic and socioeconomic disparities in endocrine therapy adherence in breast cancer: a systematic review. Am J Public Health 105:e4–e15. 10.2105/AJPH.2014.302490 25905855 10.2105/AJPH.2014.302490 PMC4455526

[46] Green AK, Aviki EM, Matsoukas K et al (2018) Racial disparities in chemotherapy administration for early stage breast cancer: a systematic review and meta-analysis. Breast Cancer Res Treat 172:247–263. 10.1007/s10549-018-4909-5 30094552 10.1007/s10549-018-4909-5 PMC6958704

[47] Puthanmadhom Narayanan S, Ren D, Oesterreich S et al (2023) Effects of socioeconomic status and race on survival and treatment in metastatic breast cancer. NPJ Breast Cancer 9:90. 10.1038/s41523-023-00595-2 37914742 10.1038/s41523-023-00595-2 PMC10620133

[48] Pittell H, Calip GS, Pierre A et al (2024) Racialized economic segregation and inequities in treatment initiation and survival among patients with metastatic breast cancer. Breast Cancer Res Treat 206:411–423. 10.1007/s10549-024-07319-5 38702585 10.1007/s10549-024-07319-5 PMC11182814

[49] Hudock NL, Mani K, Khunsriraksakul C et al (2023) Future trends in incidence and long-term survival of metastatic cancer in the United States. Commun Med 3:1–7. 10.1038/s43856-023-00304-x 37244961 10.1038/s43856-023-00304-x PMC10224927

[50] Mariotto AB, Etzioni R, Hurlbert M et al (2017) Estimation of the number of women living with metastatic breast cancer in the United States. Cancer Epidemiol Biomarkers Prev 26:809–815. 10.1158/1055-9965.EPI-16-0889 28522448 10.1158/1055-9965.EPI-16-0889 PMC5833304

[51] Anwar SL, Cahyono R, Prabowo D et al (2021) Metabolic comorbidities and the association with risks of recurrent metastatic disease in breast cancer survivors. BMC Cancer 21:590. 10.1186/s12885-021-08343-0 34022845 10.1186/s12885-021-08343-0 PMC8141199

