# Supplementary material for: Tumor biology and access to care and metastatic breast cancer outcomes
Source: Breast Cancer Res Treat. 2025 Dec 26;215(2):46. doi: 10.1007/s10549-025-07881-6 (PMC12743023; doi:10.1007/s10549-025-07881-6)
Supplement: Supplementary file 1 — Supplementary file1 (DOCX 357 KB) [file 10549_2025_7881_MOESM1_ESM.docx]

**
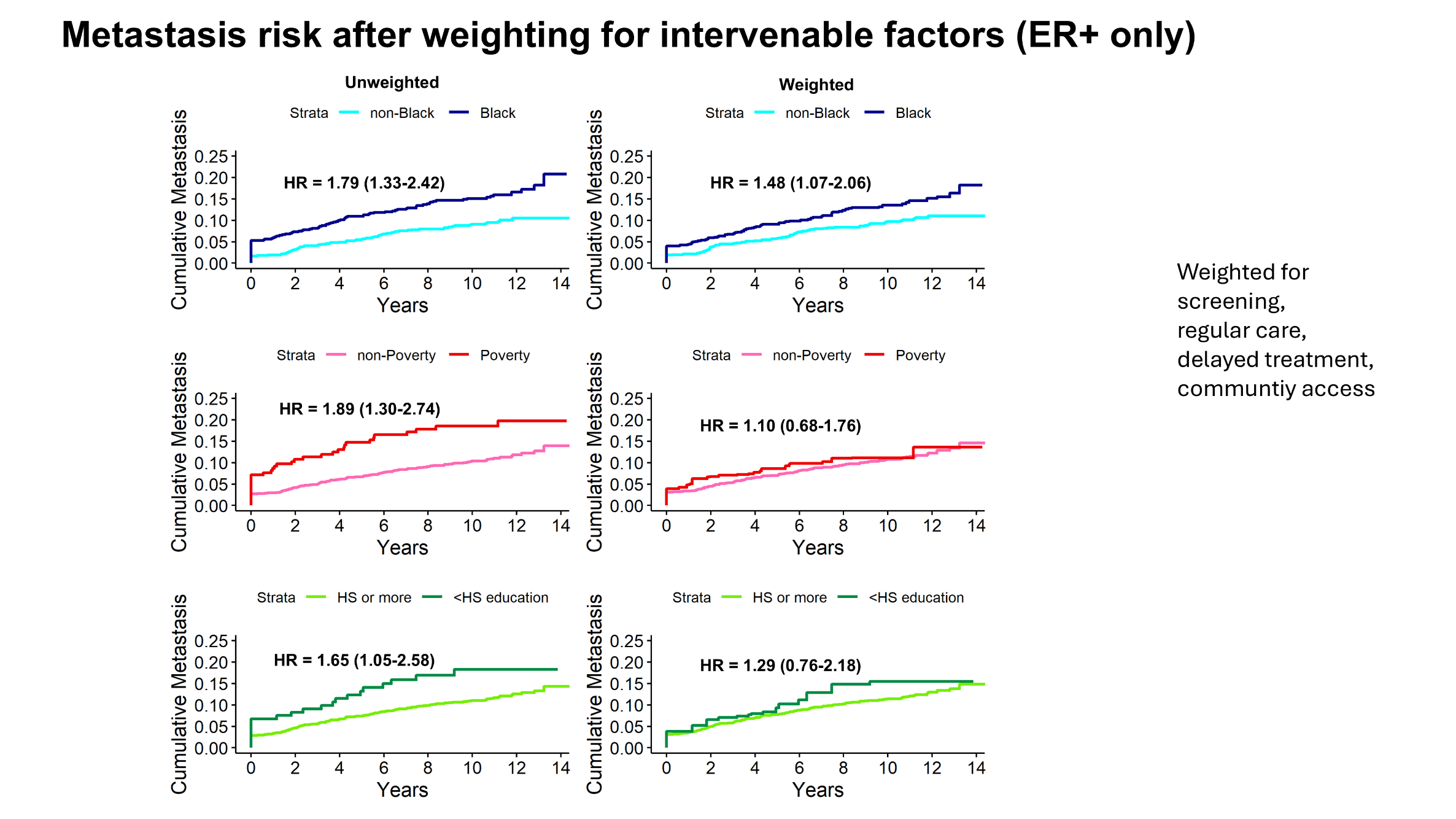
**

**Supplemental Figure 1. Cumulative incidence of metastasis with weighting for healthcare access factors (screening, regular care, delayed treatment initiation, and community healthcare access) among ER+ only.**

Cox proportional hazards models were used to model metastasis incidence using unweighted and inverse probability weighted (IPW) models with average treatment effect weights. Models were weighted for screening, regular care, delayed treatment, and community access. Analysis restricted to n=1,487 patients aged 45 years and older at diagnosis with ER+ disease.
